# *Nigella sativa* Seeds Ease Severity of Premenstrual Syndrome in Women: A Randomized, Double‐Blinded, Placebo‐Controlled Study

**DOI:** 10.1155/bmri/9811666

**Published:** 2025-11-19

**Authors:** Farjana Afrin, Mamtaz Mahal Neela, Arifin Islam, Md. Rabiul Islam, Md. Monir Hossain

**Affiliations:** ^1^ Department of Pharmacy, Jagannath University, Dhaka, Bangladesh, jagannathuniversity.org; ^2^ Department of Pharmacy, Primeasia University, Dhaka, Bangladesh, primeasia.edu.bd; ^3^ Institute of Social Welfare and Research, University of Dhaka, Dhaka, Bangladesh, du.ac.bd; ^4^ Department of Accounting & Information Systems (Statistics), Jagannath University, Dhaka, Bangladesh, jagannathuniversity.org; ^5^ School of Pharmacy, BRAC University, Dhaka, Bangladesh, bracu.ac.bd

**Keywords:** double-blinded, *Nigella sativa*, placebo-controlled study, premenstrual syndrome, premenstrual tension, randomized

## Abstract

**Background:**

Premenstrual syndrome (PMS) significantly affects women’s quality of life, with fluctuations in estrogen levels implicated in symptom severity. Lower estrogen levels during the premenstrual phase may contribute to mood swings, anxiety, and fatigue. *Nigella sativa* (*NS*) has been examined as a potential alternative therapeutic approach for various diseases. Therefore, in the current experiment, we aimed to investigate the impact of *NS* on serum estradiol levels and the severity of PMS symptoms in women.

**Methods:**

This was a randomized, double‐blind, placebo‐controlled clinical trial. Participants with moderate to severe PMS were identified using a Premenstrual Symptoms Screening Tool (PSST) questionnaire and randomly allocated to the placebo or treatment groups. Two capsules were consumed once a day for two menstrual cycles, and the severity of symptoms in the participants was monitored daily during the intervention, employing the daily record of severity of problems (DRSP).

**Results:**

At baseline, there were no significant differences (*p* > 0.05) in serum estradiol levels and total average PMS scores between the two groups. In comparison, after 2 months of *NS* supplement, serum estradiol concentrations were significantly (*p* < 0.05) increased, and the total mean PMS scores were significantly reduced when compared with the placebo group.

**Conclusion:**

The outcomes of this study indicated the favorable effects of *NS* in reducing the severity of PMS. This could be achieved by increasing serum estradiol concentration in premenstrual women with PMS. Additionally, *NS* can provide anti‐inflammatory and potential hormonal and antioxidant support, helping to reduce symptoms of PMS. Therefore, *NS* is an alternative or complementary method deserving further investigation based on scientific evidence to clarify its role in PMS treatment.

## 1. Introduction

Premenstrual syndrome (PMS) is a typical disorder in women of reproductive age manifested by a wide range of physical, psychological, and emotional symptoms starting in the luteal phase of the menstrual cycle and usually relieved by menstruation [1]. The DSM‐5 formally defines premenstrual dysphoric disorder (PMDD) as a more severe variant, requiring at least five symptoms, one of which must be a core mood symptom such as marked irritability, depression, or anxiety, that causes significant impairment in social, academic, or occupational functioning. PMS is a frequent but complex syndrome that impacts a considerable proportion of women during most of their reproductive years [1]. A large majority, approximately 95%, of women in reproductive ages are affected by PMS, which is indicative of the commonness of this health problem [2] as well as the evident heavy toll that it takes on women’s health worldwide [1, 3]. PMS may severely affect many aspects of women’s lives, such as general satisfaction and daily life performance [4].

Approaches for the management of PMS usually include a combination of lifestyle and pharmacological interventions. Standard lifestyle modifications may consist of exercise, destressing techniques, and dietary modifications (e.g., decrease intake of caffeine, alcohol, processed meals, fruits, and complex carbohydrates) [2]. In severe cases of PMS, a healthcare provider may prescribe pain medication, diuretics, antidepressants, or hormonal birth control to help relieve physical and mental symptoms [3]. Vitamin B6, calcium, vitamin D, and magnesium supplements can all be useful for PMS, but the evidence to support their use is poor [5]. Selective serotonin reuptake inhibitors are typically referred for the treatment of severe PMS and PMDD; however, their effectiveness differs among individuals, and they can have side effects [6]. Women with the severe form of PMS were found to have significantly lower levels of specific nutrients, suggesting that dietary supplementation may be a useful therapeutic approach [7]. Hormonal contraceptives may be beneficial in controlling hormone fluctuations but have side effects and are not suitable for all women [8]. Some women may not respond to standard treatments, and others may have challenging side effects, suggesting a role for further research and personalized intervention strategies [9].

Estradiol, one of the main three estrogen hormones, is the most important and dominant and is responsible for the regulation of sexual development and the menstrual cycle by stimulating the growth of the endometrium lining of the uterus in preparation for pregnancy [10]. If pregnancy does not happen, estrogen and progesterone levels decline and the lining of the uterus breaks down and is shed, along with the fluid, as menstruation. But when estrogen decreases to low levels, you get this whole host of symptoms like fatigue, bloating, cramps, headaches, anxiety, irritability, mood swings, and breast tenderness. Estrogen hormones also interact with neurotransmitters and other chemicals in the body that are involved in mood, appetite, sleep, and other bodily functions [11]. Some women experience depression and anxiety while on low levels of estrogen during the fluctuation of this hormone throughout the menstrual cycle, and, since serotonin availability in the brain can be modulated by changes in estrogen levels over the cycle, this could cause significant side effects as mood variations [12]. Women can have symptoms of altered cognition in low estrogen periods of the menstrual cycle.

There is growing evidence that oxidative stress may be involved in the etiology of PMS [13]. It has been demonstrated in clinical studies that individuals with PMS have a higher reactive oxygen species (ROS) in the blood compared with those without PMS [13]. Free radicals such as ROS induce damage to cells and tissues—that can lead to inflammation—a root factor of many of your PMS symptoms, including bloating, breast tenderness, and headaches. Furthermore, oxidative stress itself is able to raise the level of ROS, which in turn aggravates the PMS.

*Nigella sativa* (*NS*), also known as black seed, is a medicinal plant with a historical background inherited from ancient times as one member of the Ranunculaceae family, with seeds that have been applied for various pharmacological effects, scientifically explored in the last few years [14]. *NS* has numerous pharmacological effects, including effects on various systems of the human body [15]. Long‐standing use of *NS* in Middle Eastern, Indian, and North African folklore medicine has been utilized for centuries to treat an extensive variety of diseases such as asthma, bronchitis, rheumatic diseases, high blood pressure, diabetes mellitus, headache, fever, dizziness, and GI disturbances, as an antimicrobial, which illustrates the versatility of the medicinal plant [16]. Seeds of *NS* have also been used as a stimulant, diuretic, emmenagogue, lactagogue, anthelmintic, and carminative [14]. Reported pharmacological activities of *NS* are due to the presence of diverse active constituents such as thymoquinone, thymohydroquinone, dithymoquinone, thymol, carvacrol, nigellimine, nigellicine, *α*‐pinene, *β*‐pinene, d‐limonene, p‐cymene, camphene, *γ*‐terpinene, *α*‐thujene, p‐cymenene, carvacrol, and trans‐anethole [17]. Thymoquinone, in particular, has been the focus of attention as a key bioactive compound in many of the medicinal effects of *NS*. Indeed, scholarly research has reported impressive anti‐inflammatory and antioxidant potential of this herb [18]. These positive effects can be mainly ascribed to the composition of bioactive compounds such as thymoquinone, which has been shown to alleviate oxidative stress and attenuate inflammatory responses [18].

Pharmacological studies have revealed the ameliorative effects of *NS* on neuropathological disorders, diseases and dysfunctions, psychiatric outcomes, and cognitive improvement [19]. This plant has been widely used in the management of neurodegenerative aspects of Parkinson and Alzheimer disease for its substantial antioxidant potential [20]. Some research indicates *NS* may have estrogenic properties in that it binds to the body’s estrogen receptors and does some things that estrogens are known to do [21]. The *NS* includes high amounts of sex steroids such as estradiol, progesterone, testosterone, and prolactin [22]. Finally, the study suggests that *NS* may enhance estradiol level and reduce oxidative stress, indirectly suggesting possible favorable roles in postmenopausal women’s quality of life (QoL).

The prevalence of PMS is of considerable magnitude and ranges from 20% up to 98% in women during the reproductive age group, reflecting its enormous burden on the worldwide health status of women [1, 3]. Such discrepancies underscore the importance of country‐specific investigations in the precise estimation of the syndrome prevalence among populations [23]. The etiology of PMS simply continues to be an enigmatic complex puzzle, and its pathogenic mechanisms are not being fully understood, making it hard to develop research diagnostic criteria. Since the conventional treatment has some limitations and dangerous side effects, trituration is increasing in public attention as an issue for the seeking of  an alternative and complementary medicine for the treatment of PMS. Many experimental investigations have documented the positive effects of *NS,* such as its antidepressant, anti‐inflammatory, and antioxidant properties and its ability to improve memory, attention, and cognitive function. This study is designed to evaluate the effects of *NS* seeds on the severity of PMS in a randomized, double‐blind, placebo‐controlled trial.

## 2. Methods and Materials

### 2.1. Study Design and Participants

This randomized, double‐blind, placebo‐controlled clinical study was carried out among 90 participants recruited from the Dhaka region of Bangladesh. The study′s inclusion criteria specified that eligible participants were individuals aged 18–35 years with a history of regular menstrual cycles lasting 25–35 days within the preceding 6 months, who could provide informed consent and met the diagnostic requirements for PMS. Conversely, the exclusion criteria encompassed individuals who were pregnant; planning pregnancy; breastfeeding; using sex hormones; experiencing a current mood disorder (other than PMDD) or neurological condition; having an unstable thyroid or known mineral metabolism abnormality; taking medications affecting the central nervous system; consuming alcohol, tobacco, or recreational drugs; taking additional nutritional supplements; or at risk of suicide or violence. Based on these established criteria, participants were selected as the eligible study population.

### 2.2. Recruitment and Randomization

The eligible subjects were identified and recruited initially based on a screening with predefined inclusion criteria from April 1, 2024, to December 31, 2024. We used sociodemographic and reproductive questionnaires that were developed by previous studies [24]. The participants responded to a questionnaire concerning sociodemographic data, such as age, weight, height, nationality, academic background, marital status, and place of residence. Moreover, the questionnaire included questions on menstrual characteristics; the onset of first menstrual bleeding, menstrual cycle, cycle days, knowledge about the menstrual cycle, problems of going to school due to menstrual discomfort, mother/sister also have menstrual complaints, drinking frequency of coffee during the previous week, and the most frequently consumed food groups. Following that, several validated tools were used in this study to ensure an accurate diagnosis and evaluation of PMS. Initially, screening was done using the Premenstrual Symptoms Screening Tool (PSST), which provides a rapid and reliable way to determine the severity of PMS. The daily record of severity of problems (DRSP) was subsequently employed, as it is regarded as the standard of excellence for PMS monitoring and records daily variations in symptoms across two menstrual cycles. Afterward, the Beck Depression Inventory (BDI) and the Beck Anxiety Inventory (BAI) were used to screen out participants who would make it more difficult to diagnose PMS because of significant anxiety or depression. Finally, the Quality‐of‐Life Enjoyment and Satisfaction Questionnaire (Q‐LES‐Q‐SF) was used to evaluate the impact of PMS symptoms on daily life. Together, these resources ensured that participants were chosen with accuracy and that results were thoroughly evaluated.

Subjects in both groups were then given a 19‐item PSST to measure their PMS severity [25]. The PSST developers state that a positive screen is five or more self‐reported PMS symptoms, with at least one core PMS symptom that is moderately to severely affecting at least one of five realms of life. Only those who screened positive on the PSST were requested to fill out a DRSP [26] during the next menstrual cycle as it offers prospective confirmation of PMS severity. The DRSP questionnaire contains 21 items grouped into 11 domains according to the DSM‐5 criteria used to diagnose premenstrual dysphoric disorder. These items measure different types of premenstrual psychological and premenstrual physiological symptoms at different levels. This short questionnaire is another way to assess PMS since the ratings are recorded continuously throughout the menstrual cycle (not based on retrospectively reported descriptions) [27]. The DRSP utilizes a 6‐point scale to indicate symptom severity: 1—*Not at all*; 2—*Minimal*; 3—*Mild*; 4—*Moderate*; 5—*Severe*; and 6—*Extreme*. Participants are also acquainted with highlighting the days of “spotting” or “full flow of menses.” The score for each domain is determined by summing the different factors that comprise that domain, and the total DRSP score (range 21–126) is calculated by summing the individual domain scores. Participants completed the validated Persian versions of the 21‐item BDI and BAI during the mid‐follicular phase of their menstrual cycle to determine the association between their behavioral symptoms and cyclical PMS. Those with a Beck inventory score of 29 or higher were diagnosed with serious depression and severe anxiety and subsequently excluded from the study. The remaining participants were then asked to complete the DRSP during a second menstrual cycle. The study evaluated the potential of using DRSP scores starting from the first day of menstruation as a monitoring tool for PMS, consistent with the recommended protocol of recording daily symptom ratings over two consecutive menstrual cycles [28]. The participants also completed the Q‐LES‐Q‐SF twice, once during Days 1–2 and once during Days 11–13 of the menstrual cycle. Female students with moderate to severe PMS, as diagnosed using the DRSP, were ultimately included in the study. Following DRSP confirmation and screening with these instruments, 40 women with moderate to severe PMS were randomly assigned to one of two groups: the treatment group, which received *NS* capsules, or the placebo group, which received placebo capsules. As a result, both groups included women experiencing PMS, ensuring comparability between groups. Computer‐generated random variables were employed to divide individuals into two groups for receiving the *NS* supplement or placebo. The researchers and subjects were kept blinded to the randomization and allocation throughout the data analysis to maintain the blinding. The subjects were instructed to continue their normal diet and exercise routines during the research, and details of their specific dietary practices were recorded.

### 2.3. Estradiol Level Determination

During the initial phase of the study, blood samples were obtained on the third day of the follicular period of the menstrual cycle. Following an 8‐week regimen of *NS* and placebo, an additional blood sample was collected on the third day of the menstrual cycle. All blood analyses were conducted in a laboratory, facilitated by referral forms. Estradiol levels were determined using a competitive binding immunoassay enzyme‐linked immunosorbent assay kit, which enables the quantitative assessment of 17‐beta estradiol [29].

### 2.4. Preparation of *NS* Capsules

High‐quality *NS* seeds were acquired from a regional market in Dhaka. The seeds underwent thorough rinsing with clear water and complete drying. The dried seeds were then ground into a fine powder using an electric grinder. Manually, 500 mg hard gelatin capsules (Size #0) were filled with the *NS* powder. Each glass bottle contained 60 such capsules, all of which were stored at room temperature. The capsules in each bottle were unnamed, uniquely coded, and uniform in shape and packaging. The seed quality was verified through direct observation. A research team led by Dr. Md. Monir Hossain personally procured the seeds from a reputed vendor. Botanist A.M.M. Golam Adam, Professor of the Department of Botany at Jagannath University, confirmed the identity of the seeds. The capsules were manufactured in a local pharmaceutical facility compliant with Good Manufacturing Practices. Isabgol husk (psyllium seed) was similarly encapsulated in firm gelatin shells to serve as a placebo.

### 2.5. Intervention

Throughout the intervention, each participant ingested one capsule of either *NS* or a placebo twice daily for two consecutive menstrual cycles, while the control group received the placebo only. Symptom intensity was assessed during this period. The placebo and *NS* capsules were indistinguishable in appearance, odor, shape, texture, color, and size. The encoded capsules and the encryption legends were provided to the research group after the intervention was completed. This design ensured that both the participants and investigators remained blinded to the medication type during the study.

### 2.6. Study Procedures

Prior to commencing the *NS* and placebo intervention, a 5‐mL blood sample was collected from each participant following the completion of the baseline screening assessment, with the purpose of measuring their estrogen levels. On the first day of their initial cycle, study subjects began taking the daily tablets for two consecutive cycles (i.e., stopped it at the start of cycle 3). After initiating the intervention, participants completed the PSST questionnaire at the start of the first cycle based on the last preintervention cycle (Cycle 0), and then for the subsequent two consecutive cycles early in the next cycle. A follow‐up blood draw was conducted 2 months later to determine the participants’ estrogen levels after the intervention with the *NS* and placebo groups. To maintain adherence, participants were instructed to take one capsule after breakfast and another after dinner, with guidance to promptly compensate for any missed doses. Throughout the study, a research team member monitored the tablet usage and any potential complications, as well as assessed participants’ compliance with the treatment. Each subject was provided a compliance tabulated sheet and asked to record ‘yes’ after consuming their routine dose. Furthermore, the compliance sheet was evaluated regularly, reducing the possibility of missing doses for more than 1 day.

### 2.7. Statistical Analysis

This study utilized the intent‐to‐treat principle for all analyses. Demographics were compared for the *NS* group and placebo group. PMS scores were presented as mean ± SD, whereas 95% confidence intervals were computed between pre‐and postintervention periods within and between the groups. Paired sample *t*‐tests were used to examine the influence of *NS* and placebo on estrogen within pre‐ and postintervention periods (placebo and treatment groups) and *t*‐test between groups before and after intervention sessions. Furthermore, nonparametric Wilcoxon signed rank test and Mann–Whitney *U* test were used to validate the findings resulted from the paired sample *t*‐test and the independent sample *t*‐test, respectively. Statistical analyses were conducted in IBM SPSS Statistics 25 and the alpha level was *p* < 0.05.

## 3. Results

**Figure 1:**
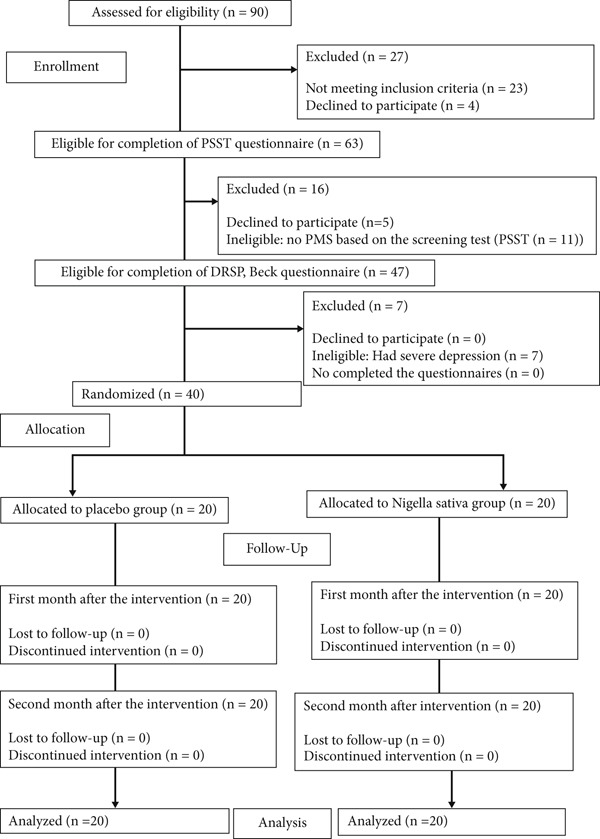
Flow diagram of the study.

A total of 23 out of 90 eligible participants did not meet the inclusion criteria, and four participants refused to participate. Following the exclusion of other test criteria, a total of 40 women were at the end randomized in two groups of *NS* and placebo (Figure 1). All 40 participants who completed the study followed two cycles of intervention as recommended. The demographic and reproductive characteristics of the two groups did not show any significant difference, as shown in Table 1.

**Table 1:** The demographic and reproductive characteristics of the participants.

**Characteristics**	**Placebo (** **n** = 20**)** (**m** **e** **a** **n** ± **S** **D** **)**	***Nigella sativa* (** **n** = 20**)** (**m** **e** **a** **n** ± **S** **D** **)**	**Mean difference**	**95% CI for the differences**	**p** **value**
Age (year)	22.50 ± 2.25	23.10 ± 1.77	−0.60	(−1.89–0.69)	0.353
Weight (kg)	51.71 ± 11.72	51.47 ± 11.40	0.22	(−7.17–7.62)	0.951
Height (cm)	153.90 ± 11.02	158.41 ± 8.92	−4.50	(−10.93–11.91)	0.163
BMI (kg/m^2^)	22.29 ± 5.28	20.29 ± 3.96	1.71	(−1.27–4.70)	0.252
Age at menarche (year)	12.95 ± 1.17	12.70 ± 1.86	0.25	(−1.28–4.71)	0.663
Duration of cycle (day)	28.40 ± 2.68	28.35 ± 3.51	0.05	(−1.95–2.05)	0.960
Duration of menstruation (day)	5.60 ± 1.77	6.05 ± 1.14	−0.45	(−1.22–0.32)1	0.247
Interval between menses	22.75 ± 3.09	22.20 ± 3.54	0.55	(−1.58–2.68)	0.604

**Figure 2:**
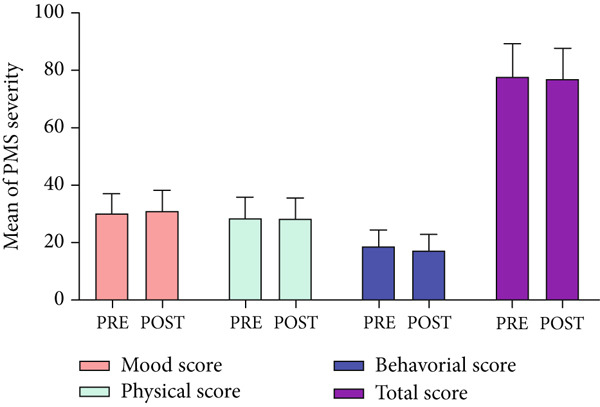
A comparative analysis of the mean PMS scores in the placebo group, examining severity in mood, physical, behavioral, and total score before (pre) and after (post) the intervention.

Total score of PMS and severity of behavioral, mood, and physical symptoms of the placebo group did not show a significant difference before and after the intervention (respectively, *p* = 0.249, *p* = 0.885, *p* = 0.148, and *p* = 0.695) as shown in Table 2. The PMS scores (mean ± SD) of 20 participants in the placebo group, examining severity in mood, physical, behavioral, and total score before (pre) the intervention were 30.18 ± 6.92, 28.51 ± 7.13, 18.71 ± 5.11, and 77.80 ± 11.45, respectively (Figure 2). After (post) 2 months of placebo treatment, the PMS scores (mean ± SD) of 20 participants in the placebo group, examining severity in mood, physical, behavioral, and total score were 31.07 ± 6.47, 28.36 ± 7.10, 17.25 ± 5.54, and 77.05 ± 10.65, respectively.

**Table 2:** Mean difference of scores of PMS symptoms within the groups (pre–post).

**Parameters**	**Mean difference (pre–post) (95% CI) within control group (** **n** = 20**)**	***p* value**	**Mean difference (pre–post)(95% CI) within *NS* group** (**n** = 20**)**	***p* value**
Mood symptoms	−0.89 (−2.46–0.67)	0.249	5.68(3.95–7.42)	< 0.001 ^∗^
Behavioral symptoms	0.15 (−2.08–2.39)	0.885	6.36 (4.60–8.12)	< 0.001 ^∗^
Physical symptoms	1.46 (−0.57–3.50)	0.148	4.26 (3.21–5.31)	< 0.001 ^∗^
Total symptoms	0.75 (−2.75–4.25)	0.695	16.20 (13.63–18.76)	< 0.001 ^∗^

^∗^Significance at 5% level of significance.

**Figure 3:**
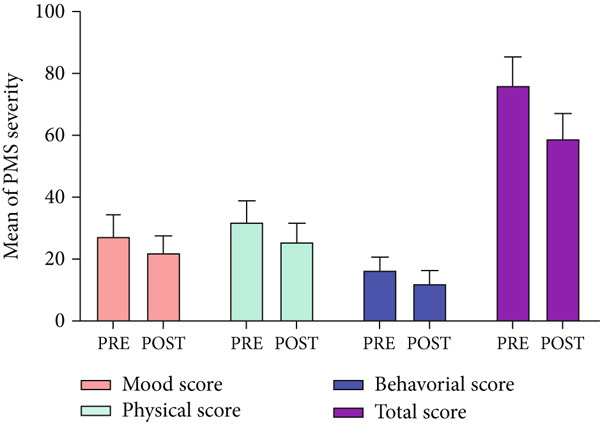
A comparative analysis of the mean PMS scores in the *Nigella sativa* group, examining severity in mood, physical, behavioral, and total score before (pre) and after (post) the intervention.

Total score of PMS and severity of behavioral, mood, and physical symptoms of the *NS* group showed significant difference before and after the intervention (all the *p* values were < 0.001) as shown in Table 2. The PMS scores (mean ± SD) of 20 participants in the *NS* group, examining severity in mood, physical, behavioral, and total score before (pre) the intervention were 27.17 ± 7.17, 31.84 ± 6.91, 16.26 ± 4.33, and 76.00 ± 9.36, respectively (Figure 3). After (post) 2 months of *NS* treatment, the PMS scores (mean ± SD) of 20 participants in the *NS* group, examining severity in mood, physical, behavioral, and total score were 21.93 ± 5.62, 25.47 ± 6.06, 11.99 ± 3.46, and 58.80 ± 8.16, respectively.

Table 2 provides the 95% confidence interval for the difference in mean scores between the pre‐ and postobservations for the mode, behavioral, physical, and total symptoms of the placebo group and the *NS* group. It has been evident that, in the placebo group (*n* = 20), mean differences (pre–post) were −0.89, 0.15, 1.46, and 0.75 for the mode, behavioral, physical, and total symptoms, respectively, and all the differences were insignificant at the 5% level of significance. On the other hand, mean differences in the *NS* group (*n* = 20) were 5.68, 6.36, 4.26, and 16.20 for the mode, behavioral, physical, and total symptoms, respectively, and all the differences were significant (*p* < 0.05) at the 5% level of significance. The mean changes in PMS scores between the two groups (placebo–*NS*) before and after the intervention are shown in Table 3.

**Table 3:** Mean difference of scores of PMS symptoms between the placebo and *NS* groups (pre–post).

**Parameters**	**Observations**	**Mean difference changes (95% CI) between groups (placebo–*NS*)**	**p** **value**
Mood symptoms	Pre	2.56 (−2.06–7.19)	0.269
	Post	9.14 (5.16–13.13)	< 0.001 ^∗^
Behavioral symptoms	Pre	2.45 (−0.66–5.56)	0.119
	Post	5.24 (2.20–8.29)	< 0.001 ^∗^
Physical symptoms	Pre	−3.32 (−7.94–1.28)	0.153
	Post	2.87 (−1.46–7.21)	0.187
Total symptoms	Pre	1.80 (−5.07–8.67)	0.599
	Post	17.25 (11.01–23.48)	< 0.001 ^∗^

^∗^Significant at 5% level of significance.

The result showed that the mean differences for mode, behavioral, physical and total symptoms before the intervention were insignificant (*p* > 0.05). After the intervention (postperiod) significant mean differences between the control and *NS* group were evident (*p* < 0.05) except for the physical symptoms (*p* > 0.05).

### 3.1. Measurement of Estrogen

**Figure 4:**
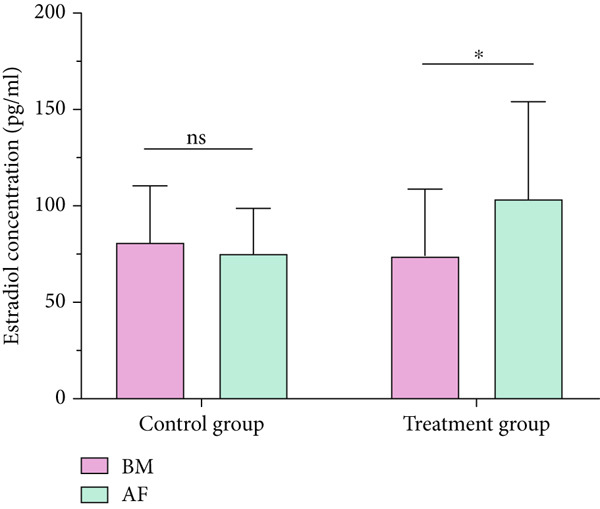
Serum estradiol levels before and after the intervention in the placebo and NS groups. BM, baseline measurement; AF, after two months (*n* = 20). Data are represented as mean ± SD, and  ^∗^ indicates that variation is statistically significant (*p* < 0.05). ns indicates that variation is not statistically significant (*p* > 0.05).

The estradiol concentrations of the placebo group and the *NS* group, measured at baseline and after 2 months, are depicted in Figure 4. This demonstrates that the concentration of estradiol in the placebo group did not differ significantly between baseline measurement (BM) and posttreatment measurement (AM), with the levels being (81.11 ± 29.37) and (75.20 ± 23.55), respectively. The *NS* group, on the other hand, exhibited a significant (p < 0.05) difference in estradiol levels between the baseline and posttreatment assessments, with levels of 103.57 ± 50.56, respectively.

### 3.2. Baseline and After‐Treatment Comparison Within the Placebo Group and *NS* Group

To determine whether the mean estradiol level for the placebo and *NS* groups exhibited a statistically significant increase from the BMs to the 2‐month time point, a paired sample *t*‐test was conducted. Null and alternative hypotheses for the placebo group was *H*_0_ : *μ*_BM_ = *μ*_AF_ and *H*_1_ : *μ*_BM_ ≠ *μ*_AF_ and for the *NS* group *H*_0_ : *μ*_BM_ ≥ *μ*_AF_ and *H*_1_ : *μ*_BM_ < *μ*_AF_  respectively, where *μ*_BM_ is the mean estrogen level for the baseline and *μ*_AF_ is the mean estrogen level after 2 months. To bolster the result of the paired sample test, Wilcoxon signed ranks test has also been applied. The results of the tests have been given in Table 4.

**Table 4:** Results of the paired sample test and Wilcoxon signed ranks test within the placebo group and *NS* group.

**Group**	**Mean difference (** **μ**_**B** **M**_ − **μ**_**A** **F**_ **)**	**Null and alternative hypotheses**	**t** **value**	**DF**	**p** **value**	**Wilcoxon signed ranks test** **p** **value**
Placebo group	5.91	*H*_0_ : *μ*_BM_ = *μ*_AF_ *H*_1_ : *μ*_BM_ ≠ *μ*_AF_	1.62	19	0.14	0.135
*NS* group	‐29.65	*H*_0_ : *μ*_BM_ ≥ *μ*_AF_ *H*_1_ : *μ*_BM_ < *μ*_AF_	−3.99	19	0.001 ^∗^	< 0.001 ^∗^

^∗^Significant at 5% level of significance.

The data presented in Table 4 indicates that for the placebo group, the test results were not statistically (*p* > 0.05) significant, suggesting no meaningful difference in the mean estradiol levels between the BM and the measurement taken after 2 months. In contrast, the statistical analyses for the *NS* group yielded significant (*p* < 0.05) results, demonstrating a substantial increase in the mean estrogen level following the administration of *NS*, in comparison to the BMs.

### 3.3. Baseline and After‐Treatment Comparison Between the Placebo Group and *NS* Group

To determine whether there was a statistically significant difference in the mean between the placebo group and the *NS* group, a two independent sample mean test was conducted. For the BMs, null and alternative hypotheses were *H*_0_ : *μ*_Control_ = *μ*_Treat_ and *H*_1_ : *μ*_Control_ ≠ *μ*_*N* *S*_, respectively, and for the measurements after 2 months, the null and alternative hypotheses were *H*_0_ : *μ*_control_ ≥ *μ*_*N* *S*_; *H*_1_ : *μ*_Control_ < *μ*_*N* *S*_, where *μ*_Control_ is the mean estrogen level for the control group and *μ*_*N* *S*_ is the mean of the estrogen for the treatment group. The results indicate that after the incorporation of the *NS*, the mean estradiol level has significantly increased compared to the placebo group. Additionally, the nonparametric Mann–Whitney *U* test was utilized to strengthen the findings of the independent sample *t*‐test. The results of these analyses are presented in Table 5.

**Table 5:** Results of the independent sample test and Mann–Whitney *U* test signed test between the placebo group and *NS* group.

**Measurements**	**Mean difference** (**μ**_**C** **o** **n** **t** **r** **o** **l**_ − **μ**_**N** **S**_ **)**	**Null and alternative hypotheses**	**t** **value**	**DF**	**p** **value**	**Mann–Whitney** **U** **test** **p** **value**
Baseline measurement	7.18	*H*_0_ : *μ*_Control_ = *μ*_Treat_ *H*_1_ : *μ*_Control_ ≠ *μ*_*N* *S*_	0.698	19	0.490	0.482
Measurement after 2 months	−28.37	*H*_0_ : *μ*_control_ ≥ *μ*_*N* *S*_ *H*_1_ : *μ*_Control_ < *μ*_*N* *S*_	−2.26	19	0.030 ^∗^	0.017 ^∗^

^∗^Significant at 5% level of significance.

There was no statistically significant difference between values from the placebo and *NS* groups at baseline, and the null hypothesis was accepted. This suggests that there was not a significant (*p* > 0.05) in mean estradiol in the baseline period in the two groups. The results of the Mann–Whitney *U* test supported the results of the independent sample *t*‐test. After 2 months, the measurements indicated a mean difference ((*μ*_Control_ − *μ*_*N* *S*_) of −28.37. Thus, to examine whether the treatment significantly increased estradiol levels, we formulated the alternative hypothesis *H*_1_ : *μ*_Control_ < *μ*_*N* *S*_. The independent samples *t*‐test revealed that the treatment had a significant (*p* > 0.05) impact on elevating estradiol levels.

## 4. Discussion

In the present study, we assessed the impact of *NS* seeds on the severity of PMS and serum estrogen levels in women with PMS in a randomized, double‐blind clinical trial manner. The results showed a significant reduction in the overall PMS score and severity of mood, physical, and behavioral symptoms’ difference in *NS* as compared with the placebo. In addition, serum estrogen was higher in the *NS*‐treated group.

There is emerging evidence that PMS may be associated with oxidative stress. It has been found that women with PMS have more ROS in their blood than those without PMS [13]. These ROS can cause harm to cells and tissues, which in turn is associated with inflammation, a contributor to PMS including bloating, breast tenderness, and headaches. In addition, oxidative stress might trigger a vicious cycle, in which ROS overproduction worsens PMS [13]. A study by Yama et al. detected more DNA oxidation prior to menstruation in women with PMS [13]. It should be affirmed that maintaining a balance between ROS generation and antioxidant defense is very important, and lower levels of antioxidant vitamins may be predictors of PMS [11]. Bioactive agents with antioxidant and anti‐inflammatory activities could also be useful to control PMS‐related complaints [30].

The antioxidant properties of *NS* may also possibly be effective in the relief of PMS [31]. *NS* and its active ingredient thymoquinone have also shown antioxidant activity [31]. These antioxidant effects can attenuate the damage from oxidative stress, which has been correlated to many health problems, including PMS [32]. Through reducing oxidative stress, *NS* might improve some physical and psychological symptoms associated with PMS [32]. It may be due also to the anti‐inflammatory properties of some of *NS* components that may participate in the possible beneficial effects of *NS* in treating PMS [33–36]. PMS may contain an inflammatory component, while *NS* and its bioactive ingredients such as thymoquinone have appeared to possess anti‐inflammatory properties in several studies. This presents a potential mechanism by which *NS* may alleviate PMS via inhibiting inflammation.

The decrease of estrogen, particularly estradiol, during premenstrual period is responsible for different PMS as estrogen regulates both the menstrual cycle and neurotransmitter function. Estrogen is known to have a relationship with neurotransmitters such as serotonin, lower levels of which can affect mood, appetite and sleep, possibly contributing to mood and cognitive changes [37]. This hormonal alteration might have a synergistic interaction with oxidative stress and inflammation, making PMS worse [38]. The question of whether *NS* influences hormone levels associated with the menstrual cycle remains an area of ongoing investigation, with some evidence suggesting a potential relationship. The sex hormones such as estradiol, progesterone, and testosterone were found in *NS* [39]. Moreover, *NS* might be useful for adjusting gonadotropins and sex hormones as evidenced by some of the reported studies which inferred that *NS* could be a strategy in controlling polycystic ovary syndrome [40]. In addition, a study of ovariectomized rats suggested that *NS* might have estrogen‐like activity [41]. These results suggest that the pathophysiology of *NS* may be related to hormonal modulation, which also may be associated with its effect on PMS, although additional studies are necessary to explore this relationship.

PMS is a complex and heterogeneous condition defined by recurrent physical, psychological, and behavioral symptoms occurring in the luteal phase of the menstrual cycle, affecting a significant part of reproductive women and reducing individuals′ QoL [2, 3]. The etiology of PMS is still an intricate multidimensional puzzle, and its pathophysiology is poorly understood. Unlike SSRIs or hormonal treatments, this natural product, *NS*, may represent an interesting therapeutic alternative with a less extreme side effect [42]. *NS* has been traditionally used for many years with few documented side effects [43]. Second, *NS* has many points of intervention, affecting inflammation, may modulate hormones, and is an antioxidant [36, 40].

Considering the constraints of the present study, more research with larger samples and longer follow‐ups is needed to consolidate the evidence of *NS* in the treatment of PMS [44]. Although several studies have indicated possible effects, larger clinical trials may help verify these findings and establish the dosage, length, and form of *NS* for the treatment of PMS signs. Further studies need to compare *NS* with conventional medications to evaluate its effectiveness and find out the target population for these therapies. In addition, the long‐term safety and effectiveness of *NS* in PMS treatment need to be investigated.

## 5. Conclusion

In conclusion, *NS* appears to have a beneficial yet exploratory role in the treatment of PMS and elevates serum estrogen levels. Preliminary evidence indicates that *NS* could exert anti‐inflammatory, putative hormonal, and antioxidant effects that may improve PMS. So, as emphasized, other data are required to understand completely the mode of action and to optimize the treatment approach. Although it has been used traditionally for different medicinal purposes and could potentially be associated with a more tolerable side effect profile than the usual treatments, validation of its effectiveness and safety in proper randomized clinical trials with large sample sizes and longer time frames is needed. Accordingly, *NS* could be a potential complementary or alternative modality that requires further scientific attention to be clarified in the management of PMS.

## Ethics Statement

The research protocol was approved by the Research Ethics Committee of Jagannath University (JnU/ERC/2024/13). Subjects were apprised of the purpose of the study, the procedures, the potential benefits and risks, and their legal right to withdraw from the study at any time. We carried out this investigation following the Helsinki Declaration’s guidelines and principles.

## Consent

The authors have nothing to report.

## Conflicts of Interest

The authors declare no conflicts of interest.

## Author Contributions

Farjana Afrin, Mamtaz Mahal Neela, and Arifin Islam: conceptualization, data curation, formal analysis, investigation, methodology, and writing—original draft. Md. Rabiul Islam and Md. Monir Hossain: conceptualization, methodology, project administration, supervision, writing—review and editing.

## Funding

No funding was received for this manuscript.

## Data Availability

The data that support the findings of this study are available from the corresponding author upon reasonable request.
